# 改良POG 9404方案治疗4例青少年初治T淋巴母细胞白血病/淋巴瘤远期疗效分析

**DOI:** 10.3760/cma.j.issn.0253-2727.2023.03.014

**Published:** 2023-03

**Authors:** 冉 赵, 燕燕 刘, 胜胜 吴, 星辰 刘, 晓旭 田, 可树 周

**Affiliations:** 1 郑州大学附属肿瘤医院，河南省肿瘤医院血液科，郑州 450003 Department of Hematology, Affiliated Cancer Hospital of Zhengzhou University and Henan Cancer Hospital, Zhengzhou 450003, China; 2 驻马店市中心医院血液科，驻马店 463000 Department of Hematology, Zhumadian Central Hospital, Zhumadian 463000, China

T淋巴母细胞白血病/淋巴瘤（T-ALL/LBL）是相对少见、恶性程度很高的血液系统肿瘤，常伴纵隔肿块和中枢神经系统（CNS）浸润，预后差，年龄≥10岁是T-ALL/LBL的不良预后因素[1]。目前国际上仍未有统一的标准治疗方案，指南推荐急性淋巴细胞白血病（ALL）样方案诱导治疗后行异基因造血干细胞移植（allo-HSCT）[2]，但无合适供者、移植后不良反应发生率及死亡率高等因素限制了其应用。对于不能移植的患者需要探索高效且安全的方案，使其获得长期生存。POG 9404研究中含有大剂量甲氨蝶呤（HD-MTX）方案（以下简称为POG 9404方案），治疗T-ALL/LBL的5年及10年无事件生存（EFS）率分别为80.2％及78.1％[1]。有研究表明大剂量左旋门冬酰胺酶（L-ASP）和增加三联鞘内注射（TIT）次数可提高疗效，减少CNS复发。我们对POG 9404方案进行改良，增加大剂量L-ASP疗程数，并将头颅放疗（CRT）替换为强化TIT以预防CNS侵犯。4例青少年患者未进行allo-HSCT也获得长期生存，且无CNS复发。现总结改良POG 9404方案的疗效、安全性和患者的长期生存情况。

## 病例与方法

1. 病例：收集河南省肿瘤医院血液科2012年4月至2017年10月接受改良POG 9404方案治疗的T-ALL/LBL患者。

2. 诊断：根据REAL分型或2016版WHO造血与淋巴组织肿瘤分类结合免疫组化对患者进行病理学诊断。所有患者均需行骨髓细胞形态学、胸部CT检查，骨髓侵犯需行流式细胞术检测。

3. 预后危险度分组：临床危险度分型参考《中国成人急性淋巴细胞白血病诊断与治疗指南（2016年版）》[3]，分为预后好与预后差两组。

4. 治疗方案：本研究在POG 9404方案基础上做了如下调整：①原方案仅在巩固治疗期应用L-ASP共20次，本研究在诱导及维持治疗期间增加L-ASP次数，全程共28次；②原方案在巩固治疗期进行预防性CRT，本研究未对患者做预防性CRT；③原方案TIT共11次，本方案增加TIT的疗程，预后好组全程18次，预后差组全程22次。

具体改良的化疗方案如下：①诱导治疗（第1～6周）：泼尼松（Pred）：40 mg·m^−2^·d^−1^，第1～22天；长春新碱（VCR）：1.5 mg/m^2^（最大剂量2 mg），第1、8、15、22天；柔红霉素（DNR）：30 mg/m^2^，第1、2、22天（辅以右丙亚胺解救）；甲氨蝶呤（MTX）：40 mg/m^2^，第2天；L-ASP：25 000 IU/m^2^，第8、15天；TIT：第1、（8）、15、22、29、36天；HD-MTX：5 g/m^2^，第22天（辅以亚叶酸钙解救）；6-巯基嘌呤（6-MP）：50 mg·m^−2^·d^−1^，第22～36天。②巩固治疗（第7～33周，每3周为1个治疗周期）：Pred：120 mg·m^−2^·d^−1^，第1～5天；VCR：2 mg/m^2^（最大剂量2 mg），第1天；DNR：30 mg/m^2^，每3周1次（辅以右丙亚胺解救）；L-ASP：25 000 IU/m^2^，20次；TIT：第10、（16）、22、（25）、28、（34）周；HD-MTX：5 g/m^2^，第7、10、13周（辅以亚叶酸钙解救）；6-MP：50 mg·m^−2^·d^−1^，第1～14天。③维持治疗［第34～108周，达到完全缓解（CR）后每3周为1个治疗周期，维持直至2年］：Pred：120 mg·m^−2^·d^−1^，第1～5天；VCR：2 mg/m^2^（最大剂量2 mg），第1天；L-ASP：25 000 IU/m^2^，6次；MTX：30 mg/m^2^，第1、8、15天；TIT：每8周1次，共10次；6-MP：50 mg·m^−2^·d^−1^，第1～14天。

5. 疗效及不良反应评价：疗效评价按NCCN指南2016年第1版分为CR、CR伴血细胞不完全恢复（CRi）、难治性疾病、疾病进展（PD）、疾病复发[4]。总反应率（ORR）＝CR率+CRi率。按美国国立癌症研究所（NCI）标准进行不良反应评价。

6. 随访：通过查阅患者住院病历及电话随访，随访截止日期为2022年2月，中位随访6.3（4.4～9.7）年。

7. 统计学处理：计量资料用中位数（范围）表示。

## 结果

1. 临床特点：接受改良POG 9404方案治疗的4例T-ALL/LBL患者中男3例，女1例，中位年龄17.5（13～27）岁，所有患者确诊时均无CNS侵犯。患者的具体临床特征见表1。

**表1:** 4例接受改良POG 9404方案治疗的T-ALL/T-LBL患者的临床特征及疗效

例号	性别	年龄（岁）	诊断	预后危险度分组	是否纵隔侵犯	初诊时WBC>100×10^9^/L	融合基因	诱导治疗33 d疗效评价	随访时间（年）
1	男	15	T-ALL	预后差	否	是	BCR-ABL190（−）、BCR-ABL210（−）、MLL-AF4（−）	CRi	7.7
2	女	20	T-ALL	预后好	是	否	CBFB-MYH11A（−）、E2A-PBX1（−）、MLL-AF4（−）、BCR-ABL190（−）、BCR-ABL210（−）	CRi	9.7
3	男	13	T-LBL	预后好	是	否	无	CR	4.8
4	男	27	T-LBL	预后好	是	否	无	CR	4.4

**注** T-ALL：T淋巴母细胞白血病；T-LBL：T淋巴母细胞淋巴瘤；CRi：完全缓解伴血细胞不完全恢复；CR：完全缓解

2. 疗效：诱导治疗第33天时，2例达CR，2例达CRi，ORR为100％。截至2022年2月，所有患者均处于CR状态，无复发。具体疗效评价见表1。

3. 不良反应：最常见不良反应为中性粒细胞计数降低（3级1例，4级3例），多在诱导和巩固治疗期出现。余不良反应有：血小板计数降低4例（2级和4级各2例），3级贫血2例。2例患者在诱导和巩固治疗过程中出现发热性中性粒细胞减少，经对症支持治疗后好转。2例患者出现肺部感染（3级1例，2级1例），经抗菌药物治疗后好转，无治疗暂停。1例患者在治疗过程中因L-ASP过敏暂停应用2次。胃肠道不良反应主要包括恶心、口腔黏膜炎、腹泻，均为1级。3例患者在巩固或维持治疗期出现1级转氨酶升高。中位随访6.3年，未观察到心脏相关不良反应及第二肿瘤，无治疗相关死亡。

## 讨论

T-ALL/LBL是起源于未成熟T淋巴细胞前体或淋巴母细胞的肿瘤，在儿童ALL中约占15％，在成人ALL中约占25％[5]。T-ALL/LBL的治疗基于多药联合的高强度ALL样方案，虽然5年EFS率及总生存（OS）率显著提高[1],[6]–[7]，但仍有近20％的患者复发。allo-HSCT能显著提高5年OS率并降低复发率，但高昂的移植费用、无匹配供者、移植相关并发症发生率高及患者的生活质量降低限制了allo-HSCT的应用[8]–[9]。POG 9404方案治疗T-ALL/LBL使长期EFS率获益[1]，本研究组在此基础上增加大剂量L-ASP应用次数，并将CRT替换为增加次数的TIT，取得良好疗效。4例青少年T-ALL/LBL诱导治疗后均获得CR/CRi，所有患者均未接受allo-HSCT，经长期随访，均未出现疾病复发及进展、第二肿瘤、死亡等事件。

L-ASP是ALL治疗方案中的重要药物。国内ALL患者L-ASP的常用剂量为5 000～10 000 IU/m^2^，多应用于诱导及巩固治疗期，5年EFS率70％～82％，5年OS率78％[10]–[11]。IDH-ALL-91研究表明，在维持治疗期应用大剂量L-ASP（25 000 IU/m^2^每周1次，共20次）能显著提高儿童ALL的无病生存率（87.5％对78.7％，*P*＝0.03）[12]。DFCI 91-01研究表明，在强化治疗期增加L-ASP（25 000 IU/m^2^每周1次，共30次）应用次数显著提高ALL患者5年EFS率（83％）；应用L-ASP≤25次患者的5年EFS率明显低于应用L-ASP>25次的患者（73％对90％，*P*<0.01）[13]。POG 8704研究表明，T-ALL/LBL患者巩固治疗期应用大剂量L-ASP（25 000 IU/m^2^每周1次，共20次）能显著提高4年持续CR率（71％对58％，*P*<0.001）[14]。因此，本研究在POG 9404方案基础上，于诱导、巩固及维持治疗期增加大剂量L-ASP的疗程（25 000 IU/m^2^，共28次）。4例不适合行allo-HSCT的青少年T-ALL/LBL患者接受改良POG 9404方案治疗，中位随访6.3年，所有患者均处于CR状态。

T-ALL/LBL复发率高，即使进行造血干细胞移植仍有部分患者复发，尤其是CNS，因此指南推荐T-ALL/LBL进行CNS预防，方法主要包括CRT、鞘内注射治疗和系统化疗[15]。尽管CRT降低CNS复发，但不良反应如内分泌异常、第二肿瘤和神经认知功能缺陷等限制其应用[16]。研究表明，鞘内注射MTX单药与接受高强度系统治疗联合CRT在预防CNS复发中作用相当，TIT预防CNS复发的作用优于MTX单药[17]–[18]。有研究表明，无论WBC高低，对泼尼松反应良好的中危T-ALL接受18次TIT治疗较预防性CRT降低了CNS复发风险[6]。EORCT-CLG58881和58951两项试验表明，T-ALL不进行CRT亦可获得良好效果，增加TIT次数在保持低CNS复发率的同时降低骨髓复发[19]。DFCI 95-01研究表明，T-ALL强化治疗期用门冬酰胺酶（ASP）及DNR代替HD-MTX也可降低CNS复发，提高疗效，5年及10年EFS率分别为85％、79％，CNS累积复发率仅3.8％[6]。前文也提及大剂量长疗程L-ASP治疗能显著提高T-ALL/LBL疗效，降低全身复发率。POG 9404方案应用TIT及大剂量L-ASP治疗T-ALL/LBL，未报告CNS复发率，入组436例，接受和不接受HD-MTX治疗的患者共26例CNS复发[1]。上述结果表明，强化L-ASP和TIT方案能减少CNS复发。因此，本研究对POG 9404方案进行改良，在CNS白血病预防中未进行CRT，并增加TIT及大剂量L-ASP次数，4例患者长期无CNS及骨髓复发。

POG 9404方案中血液学不良反应发生率较高，诱导治疗后≥3级血液学不良反应（中性粒细胞计数降低、血小板计数降低和贫血）发生率为94.4％，≥3级感染发生率为66.2％，黏膜炎发生率为17.8％；TIT引起的神经系统不良反应（仅表现为癫痫）发生率为12.2％[1]。本研究血液学不良反应最常见，多发生于诱导和巩固治疗期，≥3级中性粒细胞计数降低4例，≥3级血小板计数降低2例，较POG 9404方案稍高，可能与本研究将诱导期出现的血液学不良反应计入其中有关。余常见不良反应为肺部感染、恶心、转氨酶升高。

T-ALL/LBL具有较强侵袭性，多疗程的强化化疗可改善患者生存。本研究观察到4例未接受allo-HSCT的青少年T-ALL/LBL应用改良POG 9404方案后获得长期CR，无CNS复发，且不良反应可控，提示改良POG 9404方案值得进一步探索。
